# Women doctors and their careers in a large university hospital in Spain at the beginning of the 21st century

**DOI:** 10.1186/s12960-015-0008-4

**Published:** 2015-03-29

**Authors:** Pilar Arrizabalaga, Rosa Abellana, Odette Viñas, Anna Merino, Carlos Ascaso

**Affiliations:** Nephrology and Transplant Department, ICNU, Hospital Clinic, Barcelona, Spain; Biomedical Investigation Institute August Pi i Sunyer (IDIBAPS), Barcelona, Spain; Department of Public Health, University of Barcelona, Barcelona, Spain; Immunology Department, CDB, Hospital Clinic, Barcelona, Spain; Hemotherapy-Hemostasis Department, CDB, Hospital Clinic, Barcelona, Spain; Servei de Nefrologia i Trasplantament Renal, Hospital Clinic, c/ Villarroel, 170, 08036 Barcelona, Spain

## Abstract

**Background:**

The feminization of medicine has risen dramatically over the past decades. The aim of this article was to compare the advance of women with that of men and determine the differences between hierarchical status and professional recognition achieved by women in medicine.

**Methods:**

A retrospective study was carried out in the Hospital Clinic Barcelona, Spain, of the period from 1996 to 2008. Data relating to temporary and permanent positions, hierarchy and career promotion achieved, specialty, age and the sex of the participants were analysed with the ANOVA test and logistic regression using the generalized estimated equation.

**Results:**

After completion of specialist training, fewer women than men doctors obtained permanent positions. The ratios between the proportions of women and men remained 1.2 for permanent non-hierarchal medical positions and below 0.2 for higher hierarchal levels. Fewer women than men with hierarchy and fewer women than men achieved the rank of consultant. Promotion to consultant and senior consultant was lower than that to senior specialist, being higher in specialties with gender parity and in masculinised specialties. On comparing the two genders using a statistical model, the probability of continuous promotion decreased with the year of the application and the age of the applicant, except in women.

**Conclusions:**

Despite the number of women training as specialists having increased to 50%, women remained in temporary positions twofold longer than men. Compared to women, men showed significant representation in hierarchal medical positions, and women showed a lower adjusted probability of internal professional promotion throughout the study period.

## Background

Over the past century, women have moved from practically exclusion from medical schools towards forming the majority of new graduates in medicine, a trend referred to as the “feminization of medicine”. As in much of the western world, since two decades ago, most European university medicine graduates have been women. Women are among over 50% of the medical workforce gravitating towards general or primary care fields, and the number of women enrolled in training specialist programmes has risen markedly. Women report less interest than men in academic careers [1]. Fewer women than men have succeeded in advancement in academic medicine with a remarkable lack of female leaders reaching the rank of full professor [2].

The term “glass ceiling” has been used to describe the circumstances of women in academia and business. It implies that recognition and rank commensurating with one’s success may be visible yet unattainable for many women. Despite the gains made by women over the last decades, this “glass ceiling” is still a component of most women’s professional careers. Glass is an apt metaphor as most of the obstacles to women’s success in professional life are invisible. The consequence is the gradual dropout rate of women, known as the “leaky pipeline phenomenon”, which refers to the insufficient number of women reaching the rank of full promotion.

Feminization of medicine and its implications has been a topic of interest in the United States of America [3,4] and Europe [5]. Over the last three decades, the studies on the generic preferences that motivate students to choose certain specialties and graduate destinations and the dilemma of motherhood that may be a potential conflict to the careers of women compared with men have mainly been carried in the United Kingdom [6,7].

From 1993 to 2008, more than 70% of the medical students at the University of Barcelona, in Catalonia (Spain), have been women. Among Spanish collegiate physicians as a whole, the proportion of women has risen to 93.4% in contrast with men who have barely increased 12.6% from 1994 to 2011 [8]. Since the 1990s, women represent over 50% of the physicians enrolled in medical and surgical specialities hospital residency programmes reaching professional maturity. Will women break the “glass ceiling” of medicine in the 21st century? The answer to this question is complex due to the lack of data available on graduates joining the medical profession and continuing their career.

We analysed the proportion of women and men holding temporary, permanent and managerial medical positions, as well as the gender ratios for each of the career promotion grades over the past 10 years from data obtained from the Hospital Clinic in Barcelona (Spain). This hospital is a 750-bed university hospital providing the highest complexity of health care to a population of 540 000 inhabitants. The organizational structure consists of medical departments grouped into nine clinical institutes and two supporting centres for general biological testing and diagnostic imaging. Permanent medical or staff positions account for two thirds of the medical workforce, whereas specialist hospital trainees and temporary medical specialists make up the remaining non-permanent positions. Hierarchical medical positions are limited to the grade of medical staff. Institute and department chairs are the most distinguished positions in the medical hierarchy. Since 1996, the Professional Career (PC) system has allowed the promotion of medical staff as well as the promotion of executive management hierarchal positions. The total number of PC promotions available is limited to a given number per year. Advancement to the successive four grades (specialist, senior specialist, consultant and senior consultant) in PC promotion is obtained after evaluation of the candidate based on rules regarding clinical, research, teaching, continuous training activities, participation in corporate activities and peer opinion. Both hierarchical promotion and the promotion by the PC system are linked to the attainment of higher salaries.

We analysed the association between several factors and recruitment, hierarchical promotion and promotion of the PC system focusing on the role of sex, age, nature of the speciality and the probability of progression. This article evaluates the advance of women in comparison with men in medicine and determines whether the advance in hierarchical status and professional recognition achieved by women differs. We analysed the disparity between women and men physicians along the formal hierarchal promotion process and the recognition of professional career promotion of the physicians from 1996 to 2008.

## Methods

### Study population

A retrospective longitudinal descriptive study was designed. Data related to the medical workforce was obtained from the Human Resources Department of the hospital*.* All physicians working at this hospital in October 1996 were included evaluating information based on sex, age, medical specialty and professional status. The study included 695 physicians, 216 of whom were women and 479 men. Those joining the workforce from 1996 to 2008, were also included thereby raising the number of physicians studied to 1 135—492 women and 643 men—in 2008.

### Variables

The following variables were studied in relation to the type of employment status: permanent, temporary and training medical position, and within permanent medical positions, data relating to the category of executive management hierarchy (head of section or unit, department and institute chairs) and the grade attained on PC promotion (specialist, senior specialist, consultant and senior consultant) were recorded. Moreover sex, specialty and age were collected and analysed.

Leadership was the topic of most significance. For the purpose of this study, “leaders” were defined as chief executive officers or senior consultants. We assumed that the potential for support from colleagues such as mentoring and mutual information on career opportunities was likely to be associated with membership in a specialist category. The specialties were classified according to the ratio between the number of women and men, with three groups being obtained: i) feminized specialties, if the relationship was more than 1; ii) parity specialties, if the relationship was 1; iii) and masculinised specialties, if the relationship was less than 1.

### Statistical analysis

The demographic characteristics of the medical workforce by year of study were described as absolute and relative frequencies. The ratio between the percentage of women and men according to hierarchal positions and type of medical specialties were plotted using a bar graph. The chi-square and Fisher exact tests were used to compare possible differences in the proportion of hierarchal positions and the grade of PC promotion achieved by men and women. The association between age, hierarchal position and sex was analysed with the ANOVA test.

The ratio of the proportion of women and men according to the grade of PC was represented by a bar graph. Student’s *t* test was used to compare differences in the age at professional promotion between women and men doctors. To study whether the percentage of promotions with respect to the number of applications was different between men and women, the chi-square test was used. Moreover, the Mann–Whitney *U* test was performed to compare whether the number of applications of women to professional promotion was different from that of the men. The type I error was fixed at 0.05.

Logistic regression was used to determine the probability to achieve promotion under the rules of the PC system according to sex, the grade of PC applied to, age, the year of the application and specialty feminization of the applicant associated with the promotion. The logistic regression analysis also took into account the correlation among the repeated measures of the physician. The parameters of the logistic regression were estimated using the generalized estimated equation (GEE) assuming that the within-subject correlation structure is compound in symmetry.

## Results

### Recruitment

On completing medical school, the first step to achieve medical specialization involves passing the national Spanish internal medical resident (MIR) examination to thereafter acquire a training position for medical specialization. During the study period, the number of women doctors holding a MIR position at the Hospital Clinic was a slightly greater than 50%; thus, the ratio of women to men on completion of specialization was from 1.05 in 2000 to 1.14 in 2008. After completion of their specialist training, women held significantly more than double the temporary medical positions than men, with less than 50% of women compared to 70% of men doctors achieving permanent positions (*p* < 0.001). There was no significant difference between the mean of age of men (47.25 years (95% CI (confidence interval), 46.43 to 48.07)) and women (44.83 years (95% CI, 44.67 to 46.98)) among the permanent non-hierarchal medical positions.

Most permanent medical positions among hospital doctors were in masculinised specialties. Seven hospital specialties at the Hospital Clinic are strongly masculinised, with 3% to 24% of these specialties being comprised of women (trauma and orthopaedic surgery (3%), general surgery and subspecialties (6%), medicine (8%), cardiology and respiratory (20%) and neurology (24%)). In some specialties, more than 45% of women held permanent medical positions such as obstetrics and gynaecology (46%), medical imaging (56%), paediatrics (60%), laboratory (clinical chemistry, microbiology, pathological anatomy, genetics and immunology), and finally, in dermatology, the medium ratio between the number of women and men doctors was 1 over the study period. Only anaesthesia and child psychiatry were strongly feminized specialties.

### Hierarchal career progression

**Figure 1:**
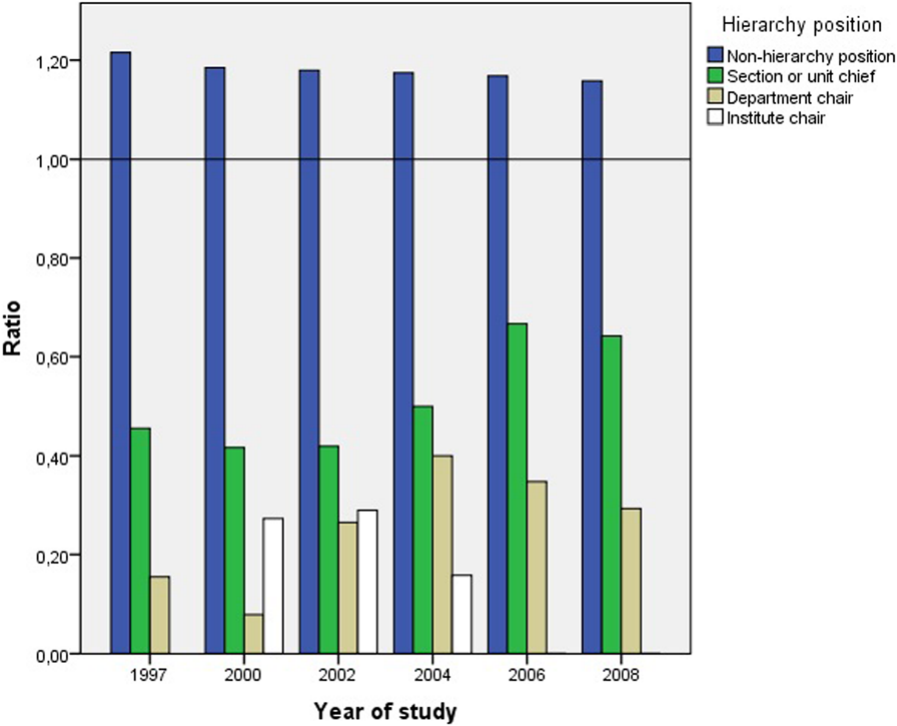
**Ratio between proportion of women and proportion of men according to the hierarchal medical positions in each year of the study.**

The ratio of 1.2 between the proportions of women and men doctors among permanent non-hierarchal medical positions decreased below 0.4 for hierarchical positions such as section or unit head and below 0.2 for institute and department chairs throughout the study period. According to data from 2006, this ratio rose slightly to 0.6 for section or unit heads, the lowest hierarchal position, but no trend to an increase was observed in any hierarchal position (*p* > 0.05) (Figure 1).

Considering the set of doctors with a hierarchical position, the proportion of women was 8.1%, 5.8%, 7.3%, 9%, 10.2% and 8.3% versus 24.4%, 20.5%, 21.4%, 22.5%, 23.3% and 20.08% in 1997, 2000, 2002, 2004, 2006 and 2008, respectively (*p* < 0.001). Thus, the proportion of women remained significantly stable at one third that of men throughout the study period.

The age of institute and department chairs to achieve promotion was 13.13 ± 0.82 (mean ± standard deviation) years greater, and that of the section or unit heads was 8.33 ± 0.66 years greater compared to doctors in permanent non-hierarchal positions (*F* = 266.53, *p* < 0.001). However, no significant differences were found regarding the age of achieving promotion according to sex among hierarchal positions.

**Figure 2:**
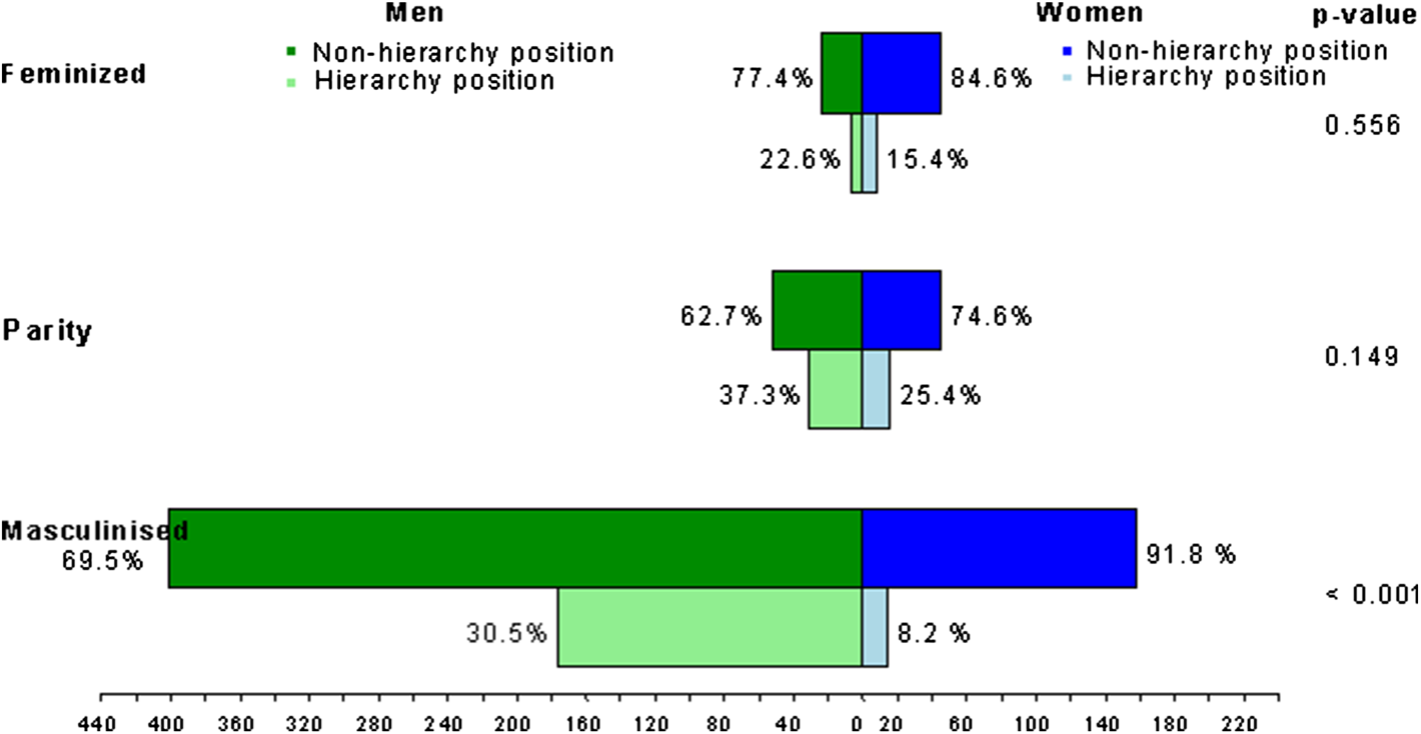
**Number and percentage of women and men doctors with and without hierarchal position according to the medical specialty.**

There was no significant difference between the proportion of women and men among hierarchal positions in feminized and parity medical specialties. However, the differences between the hierarchal positions occupied by women (8.2%) and men doctors (30.5%) were significant (*p* < 0.001) in masculinised specialties (Figure 2).

### Professional career promotion

Promotion under the PC system for women in comparison with men doctors followed a different pathway throughout the study. Men doctors progressed smoothly through the PC ranks, with almost half of the men succeeding on application for any grade of promotion. Fewer women doctors achieved the rank of consultant in comparison with men doctors, 37.4% versus 49.5% (*p* = 0.01), and this grade was achieved slightly later (95% CI, 0.0515 to 1.198). The rate of women doctors applying for senior consultant, the top level of the PC, was lower compared to men doctors, and although the percentage of success was similar, women were required to apply 1.59 times while men did so 1.3 times to attain promotion to senior consultant (*W* = 960, *p* = 0.02976) (Table 1).

**Table 1:** **Characteristics of professional career (PC) promotion at the Hospital Clínic from 1996 to 2008**

**Grade of PC**	**Gender**	**Number of applications**	**Number of promotions** ^**a**^	***p***	**Age of promotion** ^**b**^	***p***	**Number of calls** ^**c**^	***p***
Senior specialist	Men	492	267 (54.3)	0.413	45.98 (6.88)	0.936	1.68 (1.03)	0.169
	Women	286	146 (51.0)		45.87 (5.89)		1.80 (1.08)	
Consultant	Men	315	156 (49.5)	0.010	50.93 (6.00)	0.044	1.55 (0.78)	0.118
	Women	174	65 (37.4)		51.53 (5.96)		1.80 (1.11)	
Senior consultant	Men	219	108 (49.3)	0.875	56.98 (5.46)	0.068	1.30 (0.77)	0.029
	Women	48	23 (47.9)		56.18 (4.48)		1.59 (0.91)	

^a^Number of promotions and percentage between brackets.

^b^Mean and standard deviation, between brackets, of the years when they are promoted.

^c^Mean and standard deviation, between brackets, of the number of calls to which they must apply for promotion.

**Figure 3:**
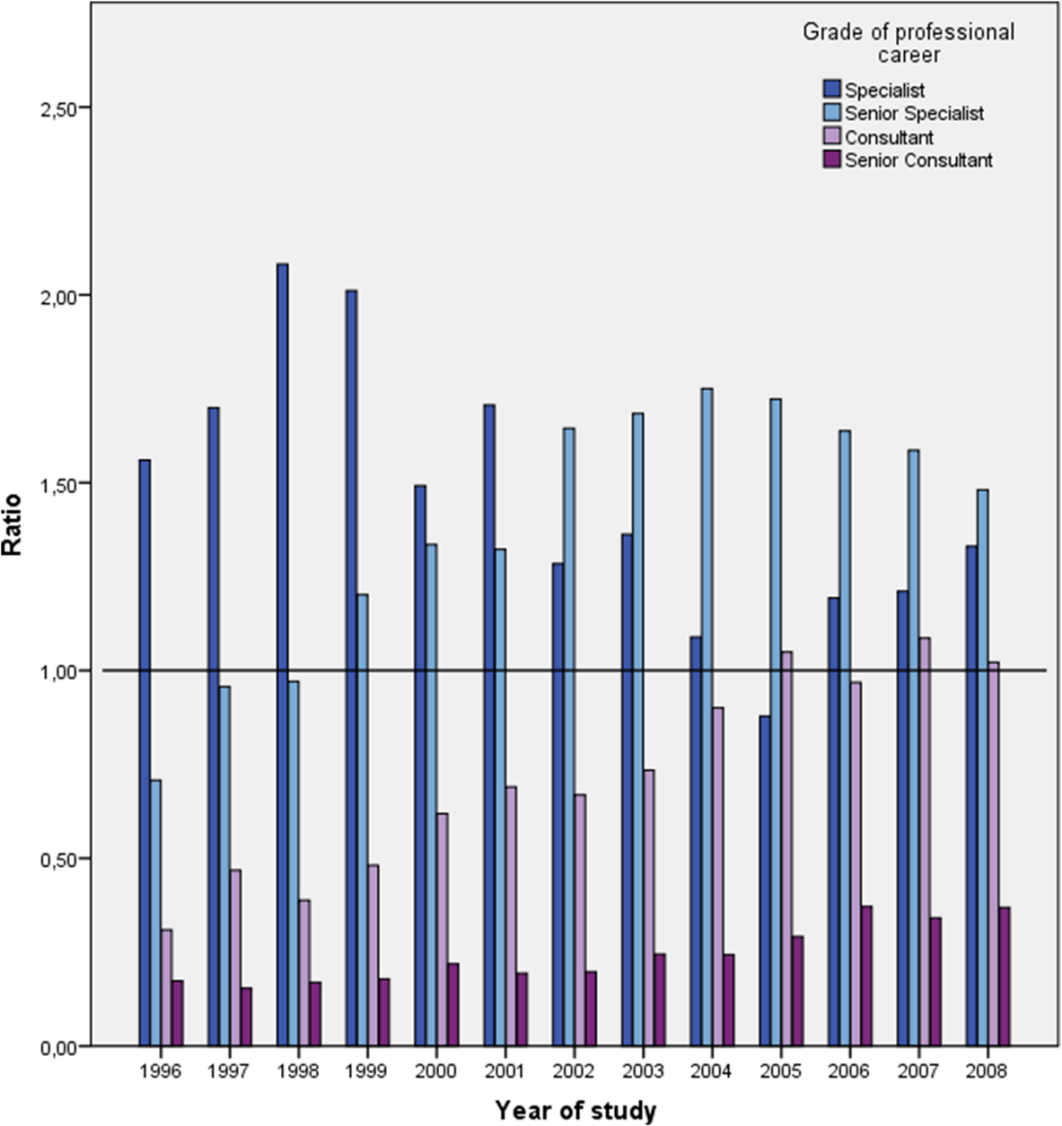
**Ratio between proportion of women doctors and proportion of men doctors according to the grade of professional career promotion in each year of the study.**

There was no significant change in the proportion of women specialists (chi-square = 0.64, *p* = 0.423) throughout the study period. On the other hand, with regard to the proportion of senior specialist (chi-square = 34.19, *p* < 0.001), consultant (chi-square = 52.29, *p* < 0.001) and senior consultant (chi-square = 12.79, *p* < 0.001) positions, the number of women increased significantly (Figure 3). However, the ratios between the proportions of women versus men among permanent medical positions remained far from the desired equity for the highest grades of PC promotion. Thus, in 2008, the last year of the study, one third of women (10.9%) compared to men (29.5%) (chi-square = 32.68, *p* < 0.0001) doctors achieved the grade of senior consultant.

On multivariate analysis, success in achieving a promotion in the PC was found to be related to sex, the grade of PC applied for, age, the year of the application and specialty feminization of the applicant (Table 2). In general, women were less likely to be promoted than men.

**Table 2:** **Variables relating to the probabilities of promotion according to the generalized estimated equation (GEE)**

**Link**	**Logit**	
**Variance to mean relation**	**Binomial**	
**Correlation structure**	**Exchangeable**	
**Parameter**	**Estimate**	**Robust standard error**
*μ*	0.955 417 895	0.255 786 186
*β*_1_	−0.222 875 922	0.135 614 955
*β*_2_	−0.064 278 247	0.412 122 598
*β*_3_	−2.020 881 817	0.665 477 787
*β*_4_	−0.605 994 396	0.037 185 707
*β*_5_	−0.398 066 411	0.011 967 823
*β*_6_	−0.385 661 872	0.272 070 952
*β*_7_	0.171 573 637	0.235 407 444
*β*_8_	0.035 426 149	0.019 260 666
*β*_9_	1.478 049 384	0.480 925 713
*β*_10_	0.102 782 104	0.740 428 923
*β*_11_	1.472 276 046	0.419 162 871
*β*_12_	0.318 933 210	0.671 786 702
*β*_13_	−0.138 421 595	0.047 412 627
*β*_14_	−0.093 635 915	0.060 024 332
*β*_15_	−0.008 454 499	0.002 762 399

Y = *μ* + *β*_1_ Women + *β*_2_ Consultant + *β*_3_ Senior Consultant + *β*_4_ Year + *β*_5_ Age + *β*_6_ Parity + *β*_7_ Masculinised + *β*_8_ Women * Age + *β*_9_ Consultant * Parity + *β*_10_ Senior Consultant * Parity + *β*_11_ Consultant * Masculinised + *β*_12_ Senior Consultant* Masculinised + *β*_13_ Consultant * Age + *β*_14_ Senior Consultant * Age + *β*_15_ Year * Age.

The probability of promotion to consultant (*β*_2_ = −0.064) and senior consultant (*β*_3_ = −2.020) was lower than that of the promotion to senior specialist. Moreover, the probability of promotion also varied according to specialty feminization. Thus, the probability of promotion was higher in the specialties with gender parity (*β*_9_ = 1.478 and *β*_10_ = 0.103) and in masculinised specialties (*β*_11_ = 1.473 and *β*_12_ = 0.318), while the probability of promotion to consultant and senior consultant was lower than the probability of promotion to senior specialist in feminized specialties. The probability of promotion decreased with the year of the application (*β*_4_ = −0.605) and the age of the applicant (*β*_5_ = −0.398) except in women (*β*_8_ = 0.035) throughout the study period. Table 3 shows the probability of promotion with average age in 2002 and the categories with the lowest predicted probability of promotion of women compared to men to the grade of consultant and senior consultant and feminized specialty.

**Table 3:** **Probabilities of PC promotion predicted by the generalized estimated equation (GEE) model with continuous variables (age and year) supposedly focused**

**Gender**	**Grade of PC**	**Specialty**	**Predicted probability**
Men	Senior specialist	Feminized	0.722 203 5
Women	Senior specialist	Feminized	0.675 362 8
Men	Consultant	Feminized	0.256 266 7
Women	Consultant	Feminized	0.216 133 9
Men	Senior consultant	Feminized	0.586 477 8
Women	Senior consultant	Feminized	0.531 594 7
Men	Senior specialist	Parity	0.635 839 5
Women	Senior specialist	Parity	0.582 847 9
Men	Consultant	Parity	0.503 629 7
Women	Consultant	Parity	0.448 098 4
Men	Senior consultant	Parity	0.513 531 5
Women	Senior consultant	Parity	0.457 915 6
Men	Senior specialist	Masculinised	0.638 706 9
Women	Senior specialist	Masculinised	0.585 860 8
Men	Consultant	Masculinised	0.505 287 4
Women	Consultant	Masculinised	0.449 738 9
Men	Senior consultant	Masculinised	0.570 206 8
Women	Senior consultant	Masculinised	0.514 950 3

## Discussion

This study shows significant differences between women and men holding permanent/staff medical positions, with differences progressively increasing in relation to the grade of advance in hierarchical promotion, and also in PC promotion. This implies that despite the proportion of women among training specialists having increased up to 50%, the bias in favour of men after completion of their specialist training could be due to the selection criteria for permanent positions. Thus, when women physicians thereafter apply for PC promotion, they have not yet obtained a permanent position, delaying the time for promotion in comparison to that of men physicians with similar years of professional experience. Using a statistical model to evaluate factors associated with the continuous career progression of medical women compared to men over a 13-year study period, the probability of promotion decreased with the year of the application and the age of the applicant, except in women. The absence of a decrease of promotion with the age in women doctors suggests that competitive women have much to offer in the second half of their careers.

The difference in permanent medical positions held by men and women is the basis of the “leaky pipeline phenomenon”, that is, a disproportionately low number of women achieve leader medical positions and advance in their careers. These results add weight to what was suggested in a previous 1-year study carried out in two Catalan hospitals [9].

There is clear evidence of secular trends in the popularity of specialties for women [10,11] to what some authors have pointed to as generic motivations and personality factors. Primary care in family medicine, obstetrics and gynaecology and paediatrics and psychiatry are all related to higher importance from the perspective scale of the measure of empathy and have been widely feminized worldwide [12]. However, the estimated probability of leadership for a 50-year-old female specialist working in hospitals in specialties such as anaesthesia, paediatrics, obstetrics and gynaecology was lower than that of their male colleagues according to a survey of 2,033 Norwegian female physicians [13]. Although the feminization of medicine involves all the medical specialties, general and digestive surgery as well as other surgical specialties continues to be chosen three times more frequently by men than by women physicians [14]. In our study, most hospital specialties registered more men than women among permanent medical positions with an even higher proportion among hierarchical positions. The proportion in favour of men among hierarchical positions is in feminized specialties and in parity specialties, with the difference being significant in masculinised specialties. This may be explained in that women do not want to assume roles in an environment where most of their colleagues are men or that there are unspoken fears that the culture of the profession is too competitive or superiors who decide promotions do not trust women and may subtly lead women far away from the medical hierarchy. A supporting network, especially in terms of a mentor, is crucial for female physicians interested in an academic career to have the opportunity to accomplish their purpose, particularly in the fields traditionally masculinised such as surgery [15].

In the United Kingdom, the proportion of women among hospital consultants represented from 12% in 1983 to 19% in 1995 and reached 25% in 2004 [16]. Our data suggest that internal promotion for women doctors has increased throughout the study, but in 2008, the last year of the study, 70% of consultants and 85% of senior consultants, the top level of the PC, were men. It has been reported that the lack of opportunities for part-time work contracts with the *British National Health Service* is associated with the lesser advance of women doctors reaching the category of consultant in comparison with men doctors [17]. Whether our doctors had full- or part-time contracts is not yet known. The law for gender equality supporting the reduction of working time to improve the work-family balance was approved in Spain at almost the end of the study period [18]; thus, we assumed that the number of part-time contracts would be negligible.

It has been argued that the dropout rate of women gradually leaving their profession rises as women mature in the medical establishment. If most European university medicine graduates have been women in the last two decades, why does the “leaky pipeline phenomenon” remain unresolved? The differences in personal values and ambitions are reportedly the reason why fewer women than men doctors hold the highest levels of medical positions. The predominant responsibility for child care is still borne by women, and the issue of balancing career and family seems to be of paramount importance among women doctors in diverse disciplines [19-21], or they believe themselves unlikely to be successful in competition. This resignation may reflect fewer women in the high echelons of medical hierarchy and the decline in the number of women applying to the top level of the PC system. Nonetheless, this hardly explains the significant fall at the second level of the PC system shown by our data. The evaluation of merits, according to the rules of the PC system, is confidential and can therefore not be discussed, but the continued difficulty in the advancement of women in comparison with their men counterparts demonstrated throughout the study period is surprising.

Do the significant disparity and lack of equity shown by our data imply that women doctors are not as good as men doctors, or do they reflect continued intangible gender barriers configuring the “glass ceiling” preventing the women from obtaining upper-level positions in hospital medicine? Since the first report from the European Technology Assessment Network in 2000 [22], the subsequent *European Guide* [23] and the more recent *European Research and Innovation* [24] show that women’s academic careers remain markedly characterized by strong vertical segregation in the field of science. In 2010, the proportion of women PhD students (38%) and PhD graduates (35%) stood at 32% of academic grade C staff, 23% of grade B and just 11% of grade A. The stable under-representation of women has suggested that subtle gender barriers act synergistically with other obstacles to career goals being achieved by women to advance to higher positions. This may be why women physicians dropout, while a small and highly competitive group of women do continue in their career progression. Gender roles contribute to unconscious assumptions that have little to do with the actual knowledge and abilities of an individual, and they negatively influence decision-making when it comes to promotions. There are differences in perceptions between the genders in relation to the “leaky pipeline phenomenon”, being attributable to subtle discrimination, and this is, suspiciously, denied by those who are well represented in scientific committees, where evaluation rules and selection mechanisms and decisions associated with candidates are made in hospital medicine.

Political government [18] and professional [25] initiatives have demonstrated that they alone are not sufficient to advance the position of women in medicine. Gender equality has an impact on the “feminization of medicine” in hospital medicine. Further evaluation of the underlying factors must be carried out by medical institutions because gender barriers are no longer accepted by women as easily as prior to the 21st century. Recognition of gender inequalities by medical institutions is the first step in helping women to advance in their careers. The gendered nature of the medical workforce worldwide means that the equality impact is quickly felt when restructuring takes place. Following this study, a new rule of the PC system emphasizing the clinical merits regarding patient care, in which women doctors are supposedly excellent [26], will be implemented in brief, hopefully improving these results in the future. The full potential of the increasing number of women physicians will not be achieved without continuing efforts to improve the ways in which they are educated and trained in becoming specialists and the mentoring women receive.

## Conclusions

Despite the number of women training as specialists having risen to up to 50%, permanent medical positions, hierarchal positions and internal professional promotion are lower compared to men. The differences in permanent medical positions held by men and women are the basis of the “leaky pipeline phenomenon”, which is still present in 21st century, and emphasize the need to review selection criteria.

Applying a statistical model of factors associated with the promotion showed that compared to men the proportion of women doctors has not stopped rising throughout the 13-year study period, despite the year of application and the age of the applicant. The absence of a decrease of promotion with the age in women doctors may play a role in the economic impact and planning of human resources for health.

## Abbreviations

PC: Professional career
GEE: Generalized estimated equation
MIR: Internal medical resident
